# Supplementary material for: m3C32 tRNA modification controls serine codon-biased mRNA translation, cell cycle, and DNA-damage response
Source: Nat Commun. 2024 Jul 10;15:5775. doi: 10.1038/s41467-024-50161-y (PMC11233606; doi:10.1038/s41467-024-50161-y)
Supplement: Supplementary file 1 — Supplementary Information [file 41467_2024_50161_MOESM1_ESM.pdf]

# $m^3C32$ tRNA modification controls serine codon-biased mRNA translation, cell cycle, and DNA-damage response

## Supplementary Information

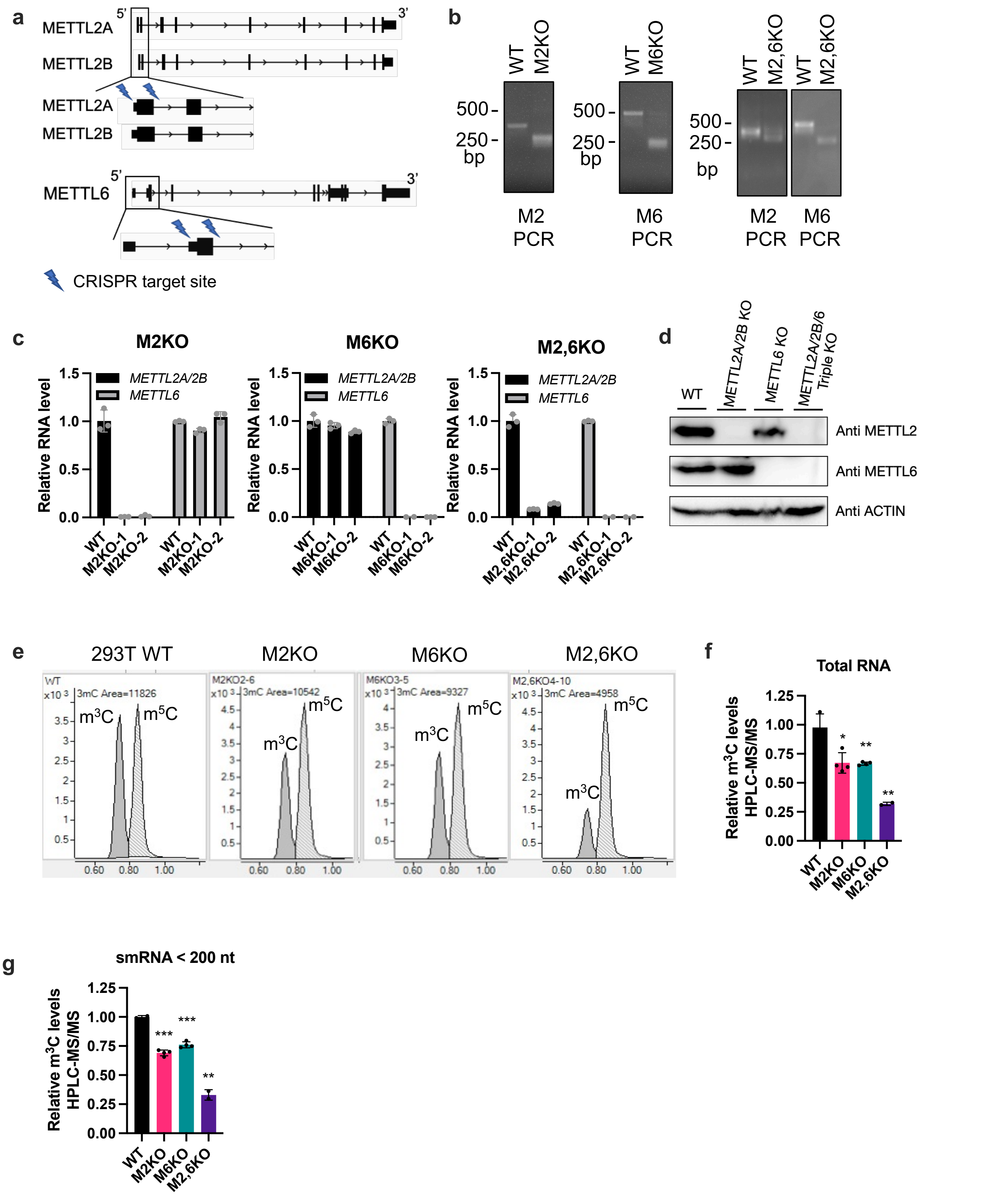


**Supplementary Figure 1. Knockout of *METTL2A/2B* and/or *METTL6* partially decreases global m$^{3}$C levels.** (**a**) Schematic diagram of CRISPR/Cas9-mediated knockout (KO) of *METTL2A/2B* and

*METTL6* in HEK293T cells. Created using IGV. (**b**) PCR validation of knockout of *METTL2A/2B* and *METTL6* using genomic DNA from WT and KO clones. (**c**) qPCR analysis of mRNA levels of *METTL2A/2B* and *METTL6* in WT and KO clones. Data shown as mean ± SD, n = 3. (**d**) Western blot analysis of HEK293T WT, *METTL2A/2B* knockout, *METTL6* knockout, and *METTL2A/2B/6* triple knockout cells (**e**) LC-MS/MS analysis of $m^3C$ and $m^5C$ on total RNA from WT and KO cells. (**f, g**) Relative $m^3C$ levels on total RNA (f) and purified small RNA (smRNA < 200 nt) (g) by LC-MS/MS analysis were compared between WT and KO cells using unmodified C as control. Data shown as mean ± SD, n = 2-4. The statistical significance was determined by unpaired student t-test where *p<0.05; **p<0.01; *** p<0.001. Source data are provided as a source data file.

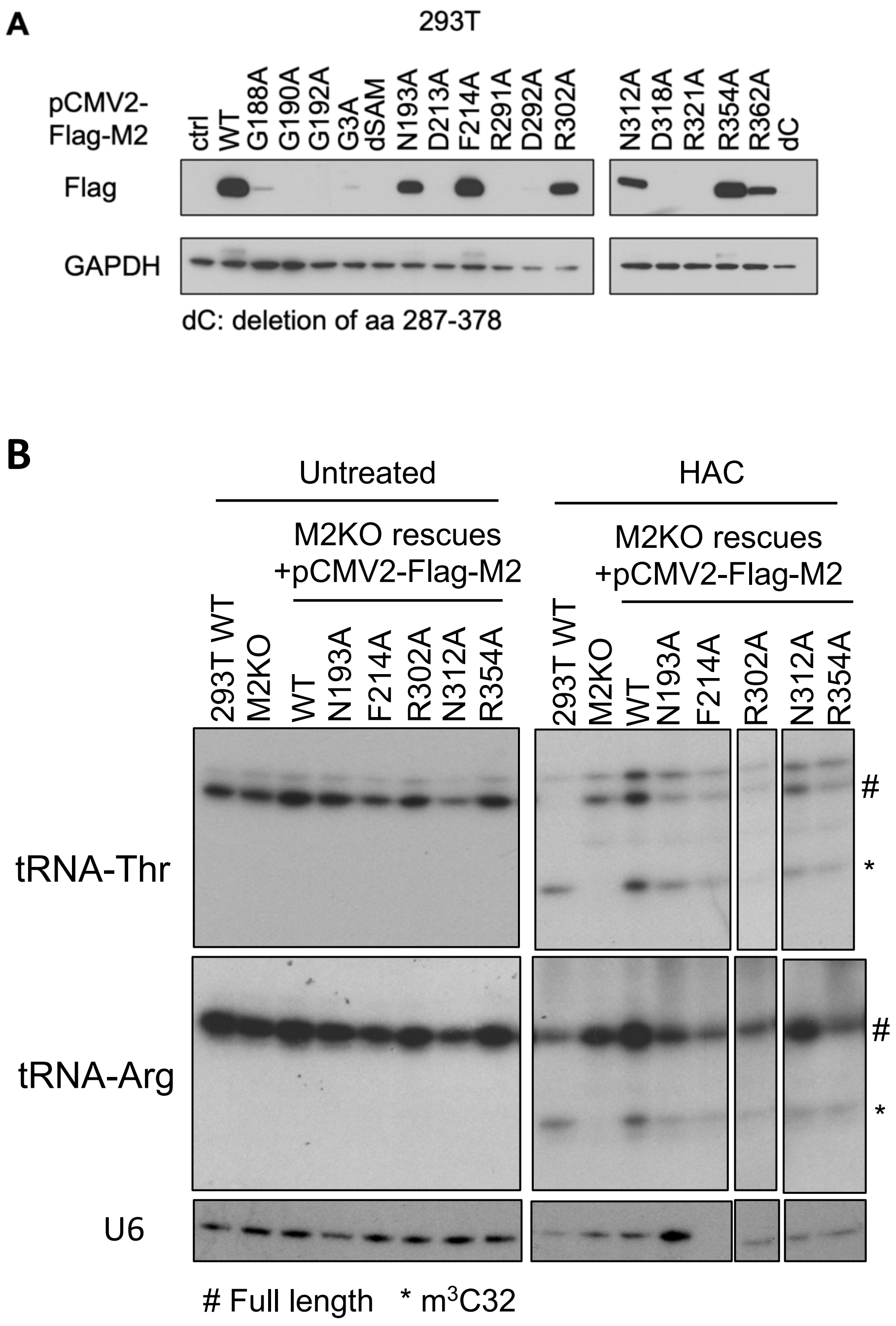


**Supplementary Figure 2. Identification of key residues required for METTL2 methyltransferase activity.** (**a**) Western blots of Flag-tagged WT and mutants of METTL2 in HEK293T cells using anti-Flag antibody. Transfection of pCMV2-Flag-empty vector was used as control (ctrl). GAPDH was used as loading control. (**b**) Northern blot detection of HAC-induced cleavage of tRNA-Arg and tRNA-Thr at $m^3C32$ showing all the highly expressed M2-WT, M2-N193A, M2-F214A, M2-R302A, M2-N312A, and

M2-R354A can rescue m$^3$C modification on tRNA in M2KO cells. U6 was used as a loading control. Source data are provided as a source data file.

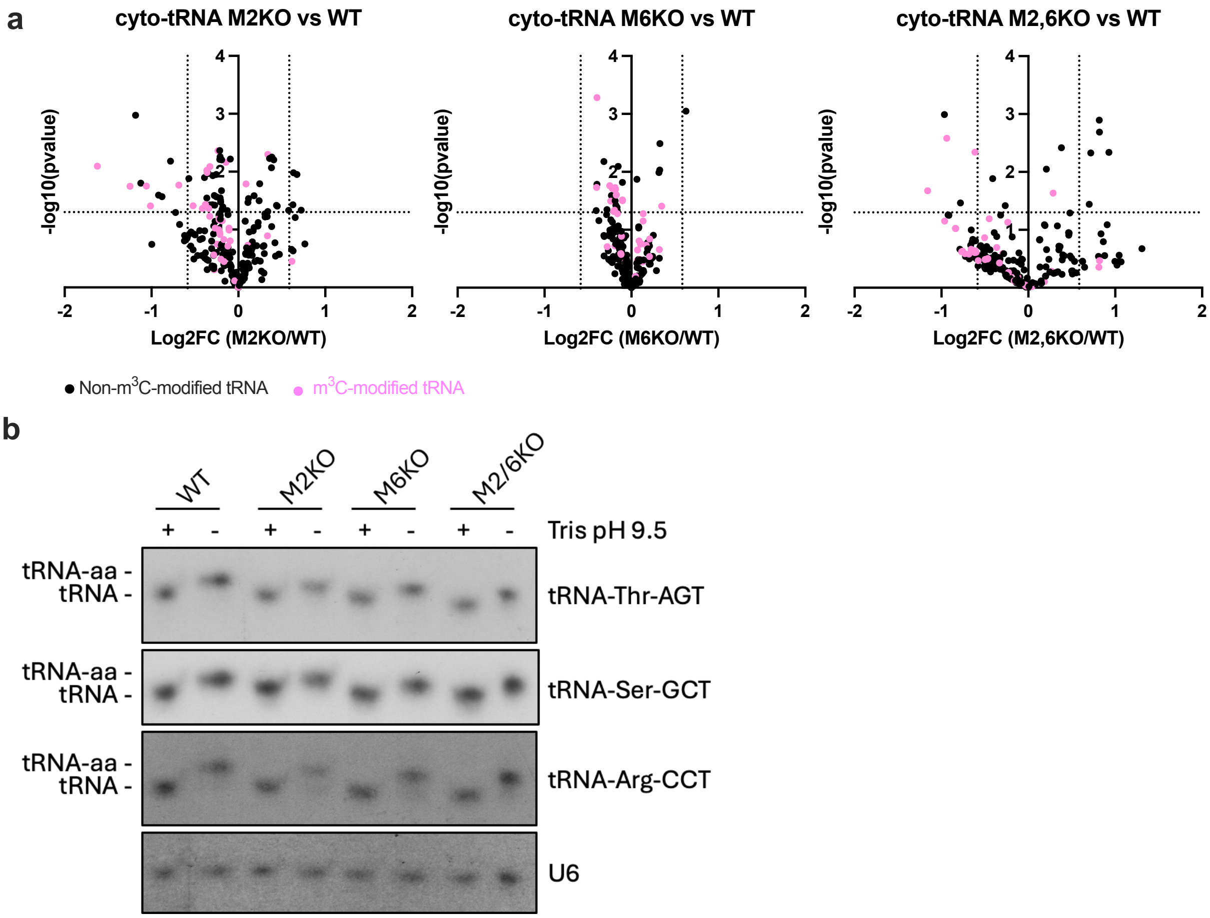


**Supplementary Figure 3. Effects of m$^3$C32 modification on tRNA expression and charging.** (**a**) Volcano plots of the fold changes (FC) in the expression levels of cyto-tRNAs upon *METTL2A/2B* knockout (M2KO), *METTL6* knockout (M6KO), or *METTL2A/2B/6* knockout (M2,6KO). (**b**) Northern blots of charged and uncharged cyto-tRNAs separated by acid-urea electrophoresis. Source data are provided as a source data file.

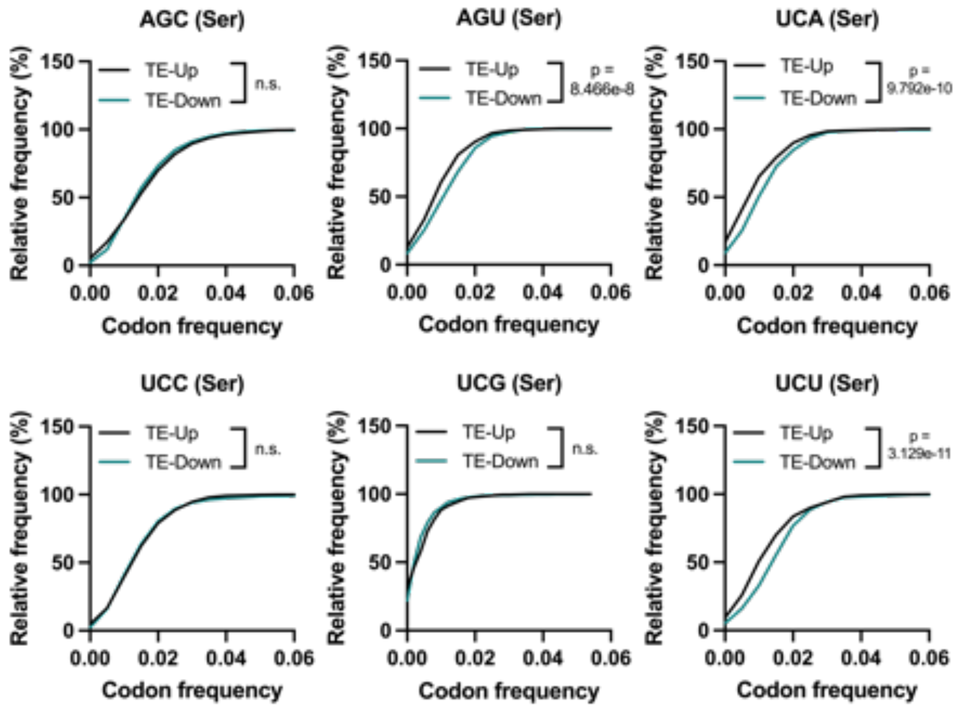


**Supplementary Figure 4. Effects of m$^3$C32 deficiency on serine codon translation.** TE: translation efficiency. p values were calculated using the two-sided Mann-Whitney test where n.s. is not significant.

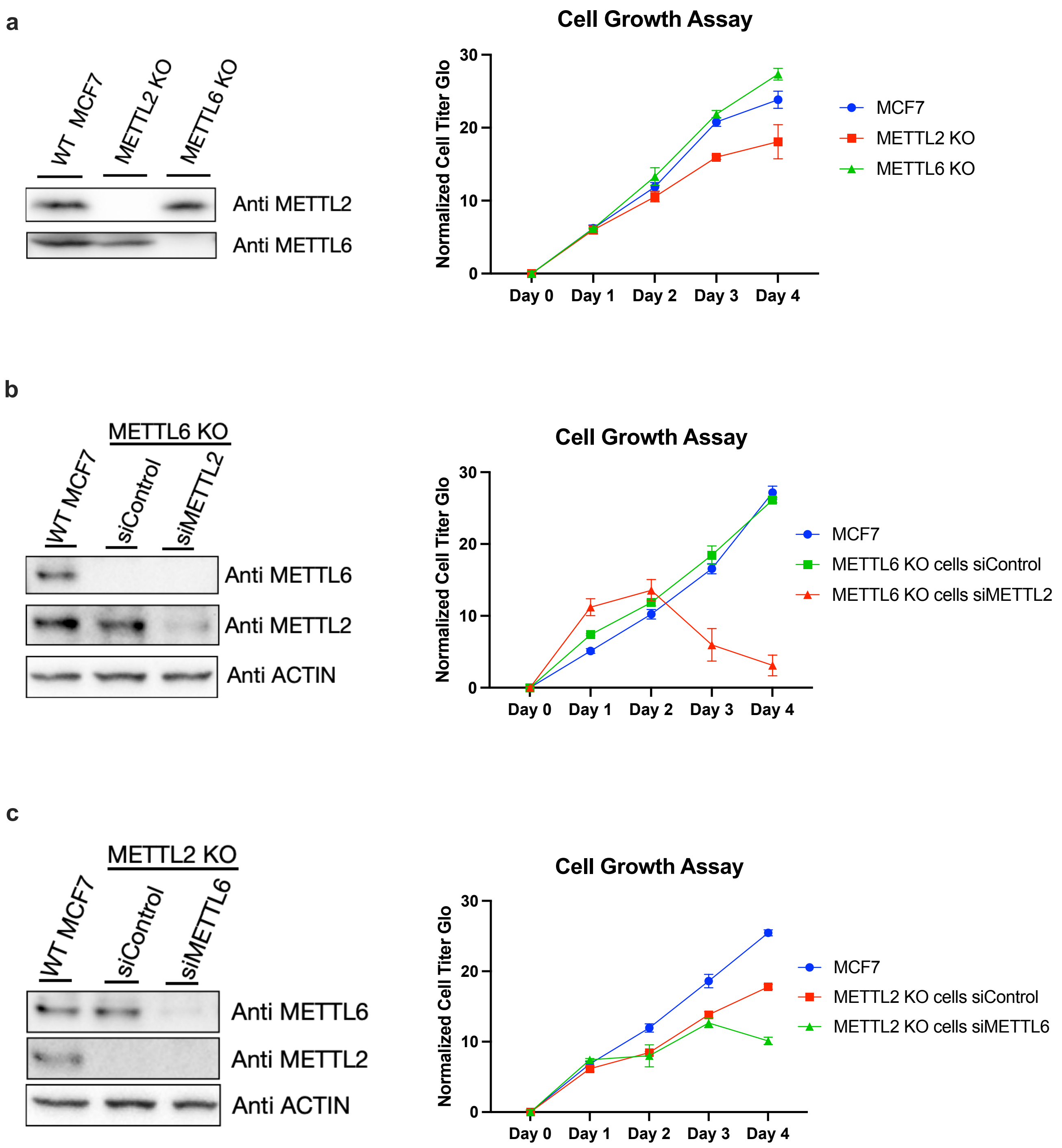


**Supplementary Figure 5. Effect of METTL2A/2B and/or METTL6 depletion on MCF7 cell growth.** (**a**) Western blot and cell proliferation assay of MCF7 WT, *METTL2A/2B*, and METTL6 knockout cells. (**b**) Western blot and cell proliferation assay of MCF7 WT, control siRNA, or siRNA-mediated METTL6 knockdown in *METTL2A/2B* knockout MCF7 cells. (**c**) Western blot and cell proliferation assay of MCF7

WT, control siRNA, or siRNA-mediated METTL2A/2B knock-down in *METTL6* knockout MCF7 cells. Source data are provided as a source data file.

## Table S1. List of primers

| Cloning Primers | | |
|---|---|---|
| pCMV2-Flag-METTL2A-WT | hMETTL2A-NotI F | AACAAGCGGCCGCGGGCTCCTACCCTGAAGGTGCAC |
| | hMETTL2A/B-WT-BglII R | AACAAAGATCTTCAGCTGGTGCTGGACAGAAGG |
| pCMV2-Flag-METTL2A-dC | hMETTL2A NotI F | AACAAGCGGCCGCGGGCTCCTACCCTGAAGGTGCAC |
| | hMETTL2A dC BglII R | AACAAAGATCTTCACCCGCCAGGTTTCAGAAGC |
| pCMV2-Flag-METTL2A-G188A | hMETTL2A G188A Q5 F | ACTGGAGGTTgccTGTGGTGTGG |
| | hMETTL2A G188A Q5 R | ATTCGGTAGGTGGCTGAG |
| pCMV2-Flag-METTL2A-G190A | hMETTL2A G190A Q5 F | GGTTGGCTGTgctGTGGGAAACA |
| | hMETTL2A G190A Q5 R | TCCAGTATTCGGTAGGTG |
| pCMV2-Flag-METTL2A-G192A | hMETTL2A G192A Q5 F | CTGTGGTGTGgcaAACACAGTCT |
| | hMETTL2A G192A Q5 R | CCAACCTCCAGTATTCGG |
| pCMV2-Flag-METTL2A-G3A | hMETTL2A G3A Q5 F | tgtggcaAACACAGTCTTTCCAATTTTAC |
| | hMETTL2A G3A Q5 R | gcacaggcAACCTCCAGTATTCGGTAG |
| pCMV2-Flag-METTL2A-dSAM | hMETTL2A dSAM Q5 F | AACACAGTCTTTCCAATTTTAC |
| | hMETTL2A dSAM Q5 R | AACCTCCAGTATTCGGTAG |
| pCMV2-Flag-METTL2A-N193A | METTL2A N193A Q5 F | TGGTGTGGGAgcCACAGTCTTTCC |
| | METTL2A N193A Q5 R | CAGCCAACCTCCAGTATTC |
| pCMV2-Flag-METTL2A-D213A | hMETTL2A D213A Q5 F | TATTGCTGTGcTTTTTCTTCCAC |
| | hMETTL2A D213A Q5 R | AACAAAGAGTCCTGGGTC |
| pCMV2-Flag-METTL2A-F214A | METTL2A F214A Q5 F | TTGCTGTGATgcTTCTTCCACAG |
| | METTL2A F214A Q5 R | TAAACAAAGAGTCCTGGG |
| pCMV2-Flag-METTL2A-R291A | hMETTL2A R291A Q5 F | GATGCTTCTGgcaGATTACGGCCG |
| | hMETTL2A R291A Q5 R | ATCCCGCCAGGTTTCAGA |
| pCMV2-Flag-METTL2A-D292A | hMETTL2A D292A Q5 F | GCTTCTGCGAgctTACGGCCGCT |
| | hMETTL2A D292A Q5 R | ATCATCCCGCCAGGTTTCAG |
| pCMV2-Flag-METTL2A-R302A | hMETTL2A R302A Q5 F | GGCTCAGCTTgcgTTTAAAAAAGGTCAG |
| | hMETTL2A R302A Q5 R | ATGTCATAGCGGCCGTAA |
| pCMV2-Flag-METTL2A-N312A | hMETTL2A N312A Q5 F | TCTATCTGGAgctTTCTACGTGAGAGG |
| | hMETTL2A N312A Q5 R | CACTGACCTTTTTTAAACC |
| pCMV2-Flag-METTL2A-D318A | hMETTL2A D318A Q5 F | CGTGAGAGGTgctGGAACCAGAG |
| | hMETTL2A D318A Q5 R | TAGAAATTTCCAGATAGACACTGAC |
| pCMV2-Flag-METTL2A-R321A | hMETTL2A R321A Q5 F | TGATGGAACCgcaGTTTACTTCTTC |
| | hMETTL2A R321A Q5 R | CCTCTCACGTAGAAATTTC |
| pCMV2-Flag-METTL2A-R354A | hMETTL2A R354A Q5 F | GCAGGTGAACgcaGGAAAGCAACTGACAATG |
| | hMETTL2A R354A Q5 R | AGTCGGCGATCCACCAGG |
| pCMV2-Flag-METTL2A-R362A | hMETTL2A R362A Q5 F | GACAATGTACgcgGTTTGGATTCAGTG |
| | hMETTL2A R362A Q5 R | AGTTGCTTTCCTCGGTTC |
| pCMV2-Flag-METTL6-WT | hMETTL6-WT-Not I F | ataagaatgcggccgcaATGGCTTCTTTGCAAAGG |
| | hMETTL6-WT-BglII-R | gaaGATCTTCAGGACTTAGGATCCAGG |
| pCMV2-Flag-METTL6 R259A | hMETTL6 R259A Q5 F | GTGTGTGCCAgcaGTTTTCCTTC |
| | hMETTL6 R259A Q5 R | AGGCCTTCTTTTTTATTCAC |
| psicheck2-6xAGC-Ser | 6xAGC-Ser Q5 F | agcagcagcGCCGATGCTAAGAACATTAAG |
| | 6xAGC-Ser Q5 R | gctgctgctCATGGTGGCTTTACCAAC |
| psicheck2-6xAGT-Ser | 6xAGT-Ser Q5 F | agtagtagtGCCGATGCTAAGAACATTAAG |
| | 6xAGT-Ser Q5 R | actactactCATGGTGGCTTTACCAAC |
| psicheck2-6xTCA-Ser | 6xTCA-Ser Q5 F | tcatcatcaGCCGATGCTAAGAACATTAAG |
| | 6xTCA-Ser Q5 R | tgatgatgaCATGGTGGCTTTACCAAC |
| psicheck2-6xTCC-Ser | 6xTCC-Ser Q5 F | tcctcctccGCCGATGCTAAGAACATTAAG |
| | 6xTCC-Ser Q5 R | ggaggaggaCATGGTGGCTTTACCAAC |
| psicheck2-6xTCG-Ser | 6xTCG-Ser Q5 F | tcgtcgtcgGCCGATGCTAAGAACATTAAG |
| | 6xTCG-Ser Q5 R | cgacgacgaCATGGTGGCTTTACCAAC |
| psicheck2-6xTCT-Ser | 6xTCT-Ser Q5 F | tcttcttctGCCGATGCTAAGAACATTAAG |
| | 6xTCT-Ser Q5 R | agaagaagaCATGGTGGCTTTACCAAC |
| psicheck2-6xGCT-Ala | 6xGCT-Ala Q5 F | gctgctgctGCCGATGCTAAGAACATTAAG |
| | 6xGCT-Ala Q5 R | agcagcagcCATGGTGGCTTTACCAAC |

| Single Guide RNAs for CRISPR-Cas9 Knockout | | |
|---|---|---|
| pX459-METTL2A/2B-KO sg 1 | hMETTL2A/B CRISPR sgRNA 1 S | CACCGTTGTGGTGGAAGACGCGCGC |
| | hMETTL2A/B CRISPR sgRNA 1 AS | AAACGCGCGCGTCTTCCACCACAAC |
| pX459-METTL2A/2B-KO sg 2 | hMETTL2A/B CRISPR sgRNA 2 S | CACCGTGTTTCCGGCTCCGGTGTCA |
| | hMETTL2A/B CRISPR sgRNA 2 AS | AAACTGACACCGGAGCCGGAAACAC |
| pX459-METTL6-KO sg 1 | hMETTL6 CRISPR sgRNA 1 S | CACCGGGAGCTAAGATCATGTAGAG |
| | hMETTL6 CRISPR sgRNA 1 AS | AAACCTCTACATGATCTTAGCTCCC |
| pX459-METTL6-KO sg 2 | hMETTL6 CRISPR sgRNA 2 S | CACCGGTTACCGTCAGTTTCAGAGA |
| | hMETTL6 CRISPR sgRNA 2 AS | AAACTCTCTGAAACTGACGGTAACC |

| | |
|---|---|
| **Guide RNA sequences used for knocking out METTL2A/2B and METTL6 in MCF7 cells** | |
| CRISPR V2 hMETTL6 guide RNA 1 forward | CACCGTCATGTACCGCCAGAGTCTG |
| CRISPR V2 hMETTL6 guide RNA 1 reverse | AAACCAGACTCTGGCGGTACATGAC |
| CRISPR V2 hMETTL6 guide RNA 2 forward | CACCGCATCTTTAGTCAGATCACAC |
| CRISPR V2 hMETTL6 guide RNA 2 reverse | AAACGTGTGATCTGACTAAAGATGC |
| CRISPR V2 hMETTL2A/2B guide RNA 1 forward | CACCGAGATTACGGCCGCTATGACA |
| CRISPR V2 hMETTL2A/2B guide RNA 1 reverse | AAACTGTCATAGCGGCCGTAATCTC |
| CRISPR V2 hMETTL2A/2B guide RNA 2 forward | CACCGCGCAGAAGCATCATCCCGCC |
| CRISPR V2 hMETTL2A/2B guide RNA 2 reverse | AAACGGCGGGATGATGCTTCTGCGC |

| | |
|---|---|
| **5' Biotin oligos for isolation of individual of tRNAs** | |
| Thr AGT oligo 1 | ACCAGCTAAGCCACGAAGCC |
| Thr AGT oligo 2 | CCTGTTTACTAGACAGGCGC |
| Thr AGT oligo 3 | CGCTGGGATTCGAACCCAGG |
| | |
| Ser GCT oligo 1 | GGAGAGGCCTGGCCGAGTGG |
| Ser GCT oligo 2 | ATGGACTGCTAATCCATTGT |
| Ser GCT oligo 3 | CTCTGCACGCGTGGGTTCGA |
