# Supplementary material for: m3C32 tRNA modification controls serine codon-biased mRNA translation, cell cycle, and DNA-damage response
Source: Nat Commun. 2024 Jul 10;15:5775. doi: 10.1038/s41467-024-50161-y (PMC11233606; doi:10.1038/s41467-024-50161-y)
Supplement: Supplementary file 3 — Reporting Summary [file 41467_2024_50161_MOESM3_ESM.pdf]

nature portfolio

Corresponding author(s): Richard Gregory

Last updated by author(s): Mar 15, 2024

Reporting Summary

Nature Portfolio wishes to improve the reproducibility of the work that we publish. This form provides structure for consistency and transparency in reporting. For further information on Nature Portfolio policies, see our Editorial Policies and the Editorial Policy Checklist.

Statistics

For all statistical analyses, confirm that the following items are present in the figure legend, table legend, main text, or Methods section.

n/a	Confirmed	
☐	☒	The exact sample size (n) for each experimental group/condition, given as a discrete number and unit of measurement
☐	☒	A statement on whether measurements were taken from distinct samples or whether the same sample was measured repeatedly
☐	☒	The statistical test(s) used AND whether they are one- or two-sided<br>*Only common tests should be described solely by name; describe more complex techniques in the Methods section.*
☒	☐	A description of all covariates tested
☒	☐	A description of any assumptions or corrections, such as tests of normality and adjustment for multiple comparisons
☐	☒	A full description of the statistical parameters including central tendency (e.g. means) or other basic estimates (e.g. regression coefficient) AND variation (e.g. standard deviation) or associated estimates of uncertainty (e.g. confidence intervals)
☐	☒	For null hypothesis testing, the test statistic (e.g. F, t, r) with confidence intervals, effect sizes, degrees of freedom and P value noted<br>*Give P values as exact values whenever suitable.*
☒	☐	For Bayesian analysis, information on the choice of priors and Markov chain Monte Carlo settings
☒	☐	For hierarchical and complex designs, identification of the appropriate level for tests and full reporting of outcomes
☐	☒	Estimates of effect sizes (e.g. Cohen's d, Pearson's r), indicating how they were calculated

Our web collection on statistics for biologists contains articles on many of the points above.

Software and code

Policy information about availability of computer code

Data collection	1. HAC-seq data was collected by using Illumina Nextseq 500 platform. 2. Ribo-seq data was collected by using Illumina Novaseq 6000 Platform. 3. HPLC-MS/MS data was collected by using Agilent Mass Hunter LC/MS Acquisition Version B.08.00. 4. qPCR data was collected by using StepOnePlus Real-Time PCR System (Applied Biosystems). 5. Flow cytometry data was collected by using BDFortessa LSRII Cell Analyzer (BD Biosciences)
Data analysis	1. For HAC-seq data analysis, after adaptor trimming and quality control, clean reads were mapped to the mature tRNA sequences downloaded from GtRNAdb using Bowtie. A maximum of two mismatches were allowed. m3C modification sites were determined by calculating the cleavage ratio at each single nucleotide on tRNA. HAC-induced cleavages of tRNA were further visualized on IGV 2.11.3. tRNA expression analysis was performed by following the ARM-seq analysis pipeline. 2. Ribo-seq data analysis was conducted using RiboToolkit (http://rnainformatics.org.cn/RiboToolkit/) . 3. HPLC-MS/MS data was analyzed by using Quantitative Analysis Version B.07.01 software. 4. All qPCR data was analyzed by using Microsoft Excel. 5. Flow cytometry data was analyzed by using FlowJo V10. 6. All statistical analysis was performed on Microsoft Excel and Graphpad Prism 9.

For manuscripts utilizing custom algorithms or software that are central to the research but not yet described in published literature, software must be made available to editors and reviewers. We strongly encourage code deposition in a community repository (e.g. GitHub). See the Nature Portfolio guidelines for submitting code & software for further information.

Data

Policy information about availability of data

All manuscripts must include a data availability statement. This statement should provide the following information, where applicable:

- Accession codes, unique identifiers, or web links for publicly available datasets
- A description of any restrictions on data availability
- For clinical datasets or third party data, please ensure that the statement adheres to our policy

High-throughput sequencing data have been deposited in the Gene Expression Omnibus (GEO) under the accession numbers GSE223418 (Ribo-Seq) and GSE223469 (HAC-Seq).

Research involving human participants, their data, or biological material

Policy information about studies with human participants or human data. See also policy information about sex, gender (identity/presentation), and sexual orientation and race, ethnicity and racism.

Reporting on sex and gender	N/A
Reporting on race, ethnicity, or other socially relevant groupings	N/A
Population characteristics	N/A
Recruitment	N/A
Ethics oversight	N/A

Note that full information on the approval of the study protocol must also be provided in the manuscript.

Field-specific reporting

Please select the one below that is the best fit for your research. If you are not sure, read the appropriate sections before making your selection.

☒ Life sciences ☐ Behavioural & social sciences ☐ Ecological, evolutionary & environmental sciences

For a reference copy of the document with all sections, see nature.com/documents/nr-reporting-summary-flat.pdf

Life sciences study design

All studies must disclose on these points even when the disclosure is negative.

Sample size	Sample size calculation was not performed. The number of samples in each experiment is based on standard practice in the field.
Data exclusions	No data was excluded.
Replication	All attempts of replication were successful. Sample sizes (n) were noted in the manuscript.
Randomization	Randomization is not applicable to our study. This study does not involve animal experiments. The samples were not used or allocated into different experimental groups. All experiments were carried out independently.
Blinding	The investigators were not blinded.

Reporting for specific materials, systems and methods

We require information from authors about some types of materials, experimental systems and methods used in many studies. Here, indicate whether each material, system or method listed is relevant to your study. If you are not sure if a list item applies to your research, read the appropriate section before selecting a response.

Materials & experimental systems

n/a	Involved in the study
☐	☒ Antibodies
☐	☒ Eukaryotic cell lines
☒	☐ Palaeontology and archaeology
☒	☐ Animals and other organisms
☒	☐ Clinical data
☒	☐ Dual use research of concern
☒	☐ Plants

Methods

n/a	Involved in the study
☒	☐ ChIP-seq
☐	☒ Flow cytometry
☒	☐ MRI-based neuroimaging

Antibodies

Antibodies used	Western blotting: anti-Flag-M2-HRP antibody (Sigma, #A8592) at a dilution of 1: 10000; anti- -actin antibody (abcam, #ab8227) at a dilution of 1: 5000; anti-GAPDH antibody (14C10, Cell Signaling Technology, #2118), anti-ATM antibody (D2E2, Cell Signaling Technology, #2873) at a dilution of 1: 1000; anti-CDC25C antibody (Proteintech, #25887-1-AP), anti-CHK1 antibody (Proteintech, #16485-1-AP) at a dilution of 1: 2000; anti-METTL2 (Proteintech Cat No. 16983-1-AP) at a dilution of 1:1000; and anti-METTL6 (Proteintech Cat No. 16527-1-AP) at a dilution of 1:1000.
Validation	The antibodies were validated by the expected band sizes on Western blots. Furthermore, some antibodies were also validated by knock-down/knock-out experiments.

Eukaryotic cell lines

Policy information about cell lines and Sex and Gender in Research

Cell line source(s)	ATCC
Authentication	Cells were not authenticated after purchasing from ATCC.
Mycoplasma contamination	Cells were tested (negative) for mycoplasma contamination.
Commonly misidentified lines (See ICLAC register)	No commonly misidentified cell lines were used.

Plants

Seed stocks	N/A
Novel plant genotypes	N/A
Authentication	N/A

Flow Cytometry

Plots

Confirm that:

☒ The axis labels state the marker and fluorochrome used (e.g. CD4-FITC).

☒ The axis scales are clearly visible. Include numbers along axes only for bottom left plot of group (a 'group' is an analysis of identical markers).

☐ All plots are contour plots with outliers or pseudocolor plots.

☒ A numerical value for number of cells or percentage (with statistics) is provided.

Methodology

Sample preparation	Cells were prepared in single cell suspension and fixed with ice-cold 70% ethanol at 4 degrees celcious for at least 1 h. Then samples were treated with 100 µg/ml RNase A (Thermo-Fisher, #EN0531) at 37 degrees celcious for 30 min followed by

incubation with 50 µg/ml PI (Invitrogen, #P3566) at room temperature for 5 min.

Instrument	BDFortessa LSRII Cell Analyzer (BD Biosciences)
Software	FACS Diva software, FlowJo software V10
Cell population abundance	300,000 cells
Gating strategy	Cells were gated first on FSC-A vs. SSC-A, then on FSC-A vs. FSC-H, and then on SSC-W vs. SSC-H. The gating strategy was applied to all the samples and the PI histogram plots were generated.

☐ Tick this box to confirm that a figure exemplifying the gating strategy is provided in the Supplementary Information.
